# Wide-field spectrally resolved quantitative fluorescence imaging system: toward neurosurgical guidance in glioma resection

**DOI:** 10.1117/1.JBO.22.11.116006

**Published:** 2017-11-01

**Authors:** Yijing Xie, Maria Thom, Michael Ebner, Victoria Wykes, Adrien Desjardins, Anna Miserocchi, Sebastien Ourselin, Andrew W. McEvoy, Tom Vercauteren

**Affiliations:** a University College London, Wellcome/EPSRC Centre for Interventional and Surgical Sciences, London, United Kingdom; b University College London, Institute of Neurology, Department of Neuropathology, London, United Kingdom; c University College London, Institute of Neurology, National Hospital for Neurology and Neurosurgery, London, United Kingdom

**Keywords:** computational imaging, multispectral imaging, glioma resection, fluorescence imaging

## Abstract

In high-grade glioma surgery, tumor resection is often guided by intraoperative fluorescence imaging. 5-aminolevulinic acid-induced protoporphyrin IX (PpIX) provides fluorescent contrast between normal brain tissue and glioma tissue, thus achieving improved tumor delineation and prolonged patient survival compared with conventional white-light-guided resection. However, commercially available fluorescence imaging systems rely solely on visual assessment of fluorescence patterns by the surgeon, which makes the resection more subjective than necessary. We developed a wide-field spectrally resolved fluorescence imaging system utilizing a Generation II scientific CMOS camera and an improved computational model for the precise reconstruction of the PpIX concentration map. In our model, the tissue’s optical properties and illumination geometry, which distort the fluorescent emission spectra, are considered. We demonstrate that the CMOS-based system can detect low PpIX concentration at short camera exposure times, while providing high-pixel resolution wide-field images. We show that total variation regularization improves the contrast-to-noise ratio of the reconstructed quantitative concentration map by approximately twofold. Quantitative comparison between the estimated PpIX concentration and tumor histopathology was also investigated to further evaluate the system.

## 1 Introduction

Primary malignant cerebral glioma, which is classified as a high-grade glioma (HGG), is identified as the most common type of primary cerebral tumor and the most fatal. Despite aggressive cyto-reductive treatment, its prognosis is poor, with an overall median survival time of ~12 months irrespective of patient age and with <5% of patients surviving longer than 5 years.^1,2^ A gross-total resection (GTR) means that the surgeon has removed all visible tumor at surgery, and no apparent tumor is identified on the postoperative MRI scans. Clinical studies have demonstrated that HGG patients who received a GTR show a significantly longer survival than those who had a subtotal resection (STR).^3,4^ For low-grade glioma (LGG) patients, GTR is also considered critical, with the five-year overall survival reported to have markedly increased from 50% to 70% in STR to 80% to 95%.^3^

Emerging intraoperative techniques have been developed and utilized in this scenario to achieve a better determination of the tumor margins. Modalities, such as intraoperative MRI,^5^ ultrasound,^6^ and optical coherence tomography (OCT),^7^ have been shown to provide structural information to differentiate brain tumor. Raman spectroscopy has been recently studied in patients during glioma resection surgery, providing spectral tissue characteristics in real time.^8^ Fluorescence imaging guidance involving 5-aminolevulinic acid-induced protoporphyrin IX (5-ALA-PpIX) as a fluorescent contrast agent has been demonstrated to provide from structural to cellular information when using camera-based wide-field imaging and probe-based laser confocal endomicroscopy (pCLE), respectively.^9,10^ The oral 5-aminolevulinic acid (5-ALA) is metabolized into fluorescence protoporphyrin IX (PpIX) molecules and preferably accumulated in glioma cells, with a ratio ranging from 10:1 to 50:1 between HGGs (grade III and grade IV) and normal brain tissue providing fluorescent contrast.^11^ Clinical studies have shown that 5-ALA-PpIX-guided excision of HGGs was capable of achieving improved complete resection rates with more than 50% GTR and prolonged patient survival compared with conventional white-light-guided resection.^9,12,13^ However, PpIX only emits very weak fluorescence at the infiltrated margins of HGGs,^14^ and similarly in LGGs,^15^ owing to the low PpIX concentration in these tissue.^15^ In these cases, it remains challenging to precisely delineate the tumor margins. Furthermore, current commercially available fluorescence imaging systems rely on visual assessment by the surgeon to distinguish fluorescence patterns, which makes the detection more subjective than necessary.

It has been demonstrated that quantitative assessment of 5-ALA-PpIX fluorescence for glioma resection can be approached using fiber-optic spectroscopy measurements.^16–18^ Among them, Kim et al.^19^ developed an intraoperative contact optical probe and a computational light propagation model to extract the absolute concentration of the fluorophore PpIX in the examined tissue, providing neurosurgeons a rough estimation of the tumor pathological degree. Similar to other probe-based measurements, such as Raman spectroscopy and pCLE, their quantification method only determines the local tissue at the contact point of the probe.^8,20^ It is of great interest, yet it remains challenging to implement PpIX quantification for wide-field images. To this end, Valdés et al.^21^ reported their approach on the estimation of PpIX concentration of a wide-field imaging system. In the estimation paradigm, local PpIX concentration is calculated in a pixel-wise manner across the whole wide-field image, based on a correction algorithm that compensates for the distortion effect of the tissue optical attenuation. Their algorithm was developed initially for a probe-based system and then directly applied to the wide-field scenario where the lighting and detection geometry were neglected. Taniguchi et al.^22^ developed a computational model that was able to extract PpIX emission from RGB images that were acquired similarly in glioma resection surgery. In the model, lighting and imaging geometry, PpIX absorption and emission spectrum, and spectral sensitivities of the RGB camera were considered to reconstruct the PpIX emission region on the wide-field image. They used their model to assess quantitative PpIX concentration on a phantom study. No tissue study was performed to evaluate this approach. In addition, the impact of the tissue optical properties in terms of absorption and scattering on retrieving the quantitative PpIX concentration was not included in their study.

In our work, we introduce a wide-field spectrally resolved quantitative fluorescence imaging modality with the objective of providing an effective computer-assisted surgical guidance for glioma resection. We adapt and expand the fluorescence model to the wide-field imaging to retrieve PpIX concentration from the emission spectrum. In our proposed approach, we calibrate the measured diffuse reflectance of the specimen to a reference diffuse reflectance where the lighting and detection geometry are specifically addressed. We deploy a high-sensitivity scientific CMOS camera to capture sufficient fluorescence signal at short exposure times, thus allowing the reduction of total data acquisition time. Furthermore, to reduce the impact of acquisition noise on the reconstructed concentration map, we apply isotropic total variation (TV) *L*1-norm regularized least-squares optimization to retrieve the PpIX concentration map over the wide-field images. The system, together with the reconstruction method, is validated in phantom experiments and in *ex vivo* human brain tumor studies. To the best of our knowledge, we performed the first qualitative and quantitative spatially localized comparison between the estimated PpIX concentration value over a wide-field image and whole-slide histopathology.^11,23,24^ The results reveal a linear correlation between our measurements and cellularity, suggesting that the proposed system is capable of quantitative delineation of tumor and could be implemented in clinical practice for assisting tumor resection.

## 2 Materials and Methods

### 2.1 Wide-Field Spectrally Resolved Quantitative Fluorescence Imaging System

**Fig. 1:**
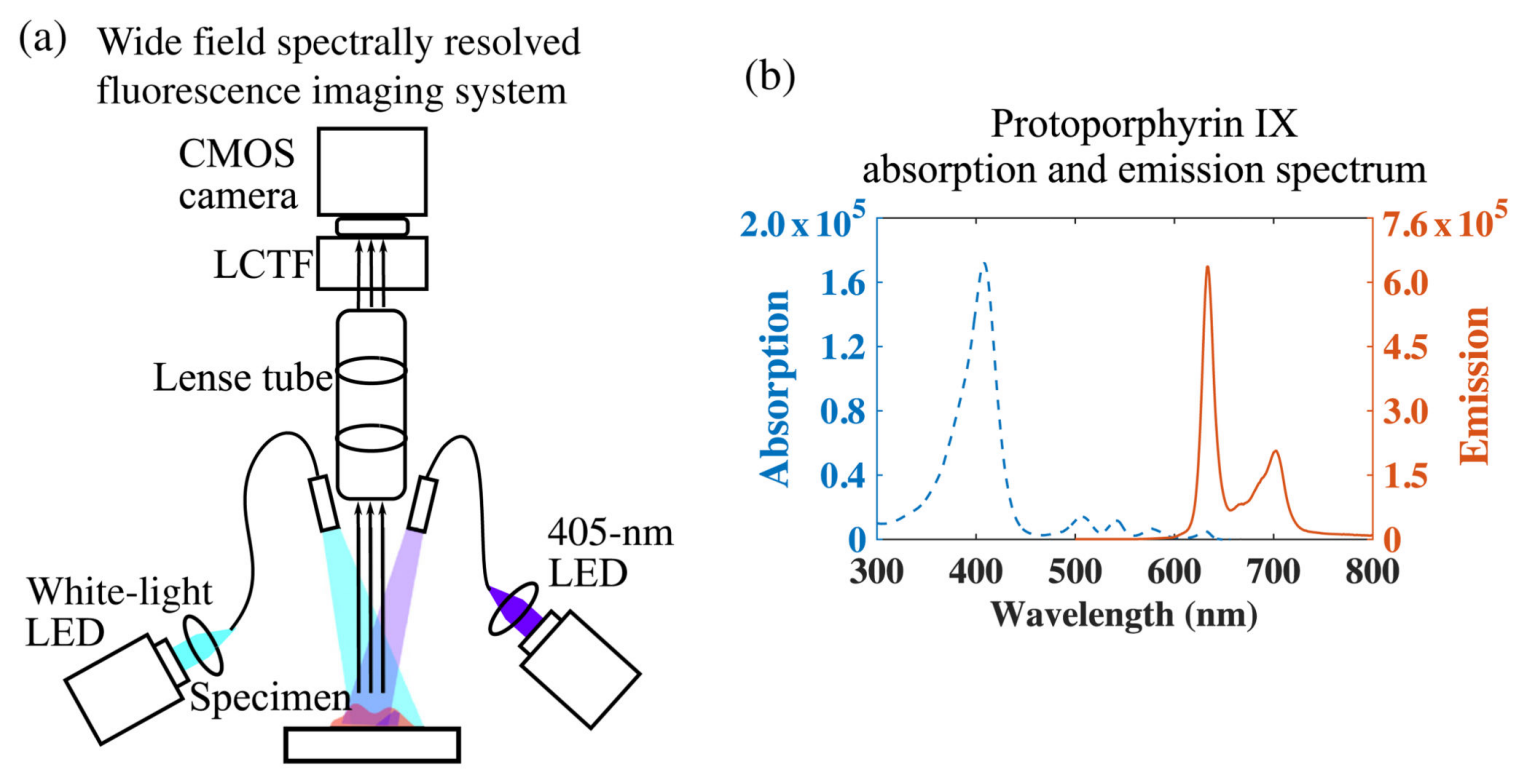
(a) Schematic of system setup and (b) the excitation and emission spectrum of PpIX (A.U.) plotted from published data.^25^ LCTF, liquid crystal tunable filter and MO, microscope objective.

The system developed in this study consists of three main modules: (1) the light sources, which include a white-light LED (MCWHF1, Thorlabs) and a 405-nm LED (M405FP1, Thorlabs), (2) a liquid crystal tunable filter (LCTF) (VariSpec-VIS, PerkinElmer), and (3) a Generation II (Gen II) CMOS camera (ORCA-Flash4.0, Hamamatsu Photonics) [shown in Fig. 1(a)]. The 405-nm LED was chosen specifically for PpIX excitation, as the peak excitation wavelength of PpIX locates at around 405 nm [Fig. 1(b)]. The LCTF performs as a bandpass filter that controllably selects a single wavelength (with 20-nm full-width at half-maximum) of light to transmit into the camera with a maximum transmittance of 27.5% of randomly polarized or unpolarized light at 710 nm. It features a spectral range from 400 to 720 nm with a tuning accuracy of 2.5 ± 0.5 nm and a tuning response time of 50 ms. The highly sensitive scientific camera (Gen II CMOS) used has low read-out noise of 1.0 electron (median) and high speed read-out (100 frame/s with a full pixel range of 2048 × 2048 pixel) compared with the state-of-the-art cameras [scientific CMOS, electron-multiplying CCD (EMCCD)] that have been used in wide-field fluorescence imaging for glioma surgery.^26,27^ The wavelength-specific images were acquired using a custom LabView (National Instruments, Austin, Texas) program, which implements hardware control, data acquisition, data storage, and data processing.

### 2.2 Data Acquisition

Fluorescence excitation is provided using a fiber-coupled 405-nm LED through a beam collimator, thus providing sufficient excitation power, whereas the diffuse reflectance excitation is implemented using a more powerful fiber-coupled white-light LED with a divergent beam. The power density of 405-nm light and broad spectrum white-light at the sample surface was measured as 1.45 and 2.00 mW/cm^2^, respectively. For both excitation schemes, three-dimensional image cubes (*x, y, λ*) were produced from successive CMOS camera frames when scanning the tuning wavelength of the LCTF across its full spectral range (400 to 720 nm) with 10-nm incrementing steps, in a manner of one frame per tuning wavelength. The exposure time was set to 100 ms for all wavelengths under both excitation scenarios to achieve a sufficient signal-to-noise ratio (SNR), except when evaluating the system performance where the exposure time was set in different values (for details, see Sec. 2.6.2). 2 × 2 pixel binning was utilized to further enhance SNR, which generated 1024 × 1024 pixels images. An additional 650-ms waiting time per frame was required in our implementation to allow saving each frame file (5 Mb/frame) to a storage media via our Labview program. In this way, each image cube contained (*x*)1024 × (*y*)1024 × (*λ*)33 spectra-specific pixels, acquired and saved in 26.4 s. For each pixel (*i, j*) in an image, the fluorescence spectrum responses using 405-nm excitation *F_i,j_*(*λ*) and diffuse reflectance spectrum using white-light excitation *R_i,j_*(*λ*) were acquired.

### 2.3 Qualitative RGB Images

We composed the wide-field white-light RGB images from the hyperspectral cubes by superimposing three distinct spectra images acquired under white-light illumination, including red 630 nm, green 540 nm, and blue 470 nm, whereas the corresponding UV light RGB images were created with spectra images that were obtained under UV illumination consisting of red 630 nm, green 540 nm, and blue 470 nm.

### 2.4 Fluorescence Quantification

In biological tissue, the fluorescence emission spectrum is distorted due to tissue’s optical absorption and scattering.^28^ The measured fluorescence emission spectrum of PpIX in brain tissue is affected, indicating it does not trivially reflect the true concentration of the PpIX, which correlates to the cancerous cellular density. In the work of Valdés et al.,^21^ the measured fluorescence spectrum was corrected based on a diffuse reflectance mode.^19^ In their correction algorithm,^21^ fluorescence spectrum *F*(*λ*) coming from the sample surface is assumed to be proportional to the diffuse reflectance *R*_em_ over the PpIX emission band [Eq. (1)]

(1) $$F(\lambda )=(1-R_{\text{ex}})\left[\frac{f_{\text{PpIX}}(\lambda )}{\mu_{a,\text{ex}}}\right]R_{\text{em}},$$

where *R*_ex_ and *R*_em_ are the diffuse reflectance at the excitation band and emission band, respectively, *f*_PpIX_(*λ*) is the ground-truth PpIX emission spectra, and *μ*_*a*,ex_ is the tissue absorption coefficient at the PpIX excitation band (405 nm). In practice, they correct the measured fluorescence spectrum *F*_meas_(*λ*) with Eq. (2); the corrected fluorescence spectrum *f*_corr_(*λ*) is then used in lieu of *F*(*λ*) and is expressed as

(2) $$f_{\text{corr}}(\lambda )=\frac{F_{\text{meas}}(\lambda )}{\Phi_{\text{ex}}\Phi_{\text{em}}^{\alpha }},$$

where Φ_ex_ and Φ_em_ are the diffuse reflectance integrated over *λ* ∈ [465,485] nm and *λ* ∈ [625,645] nm, respectively, *α* is the calibration factor determined empirically, and *μ*_*a*,ex_ is neglected (e.g., set to 1) from Eq. (1).

**Fig. 2:**
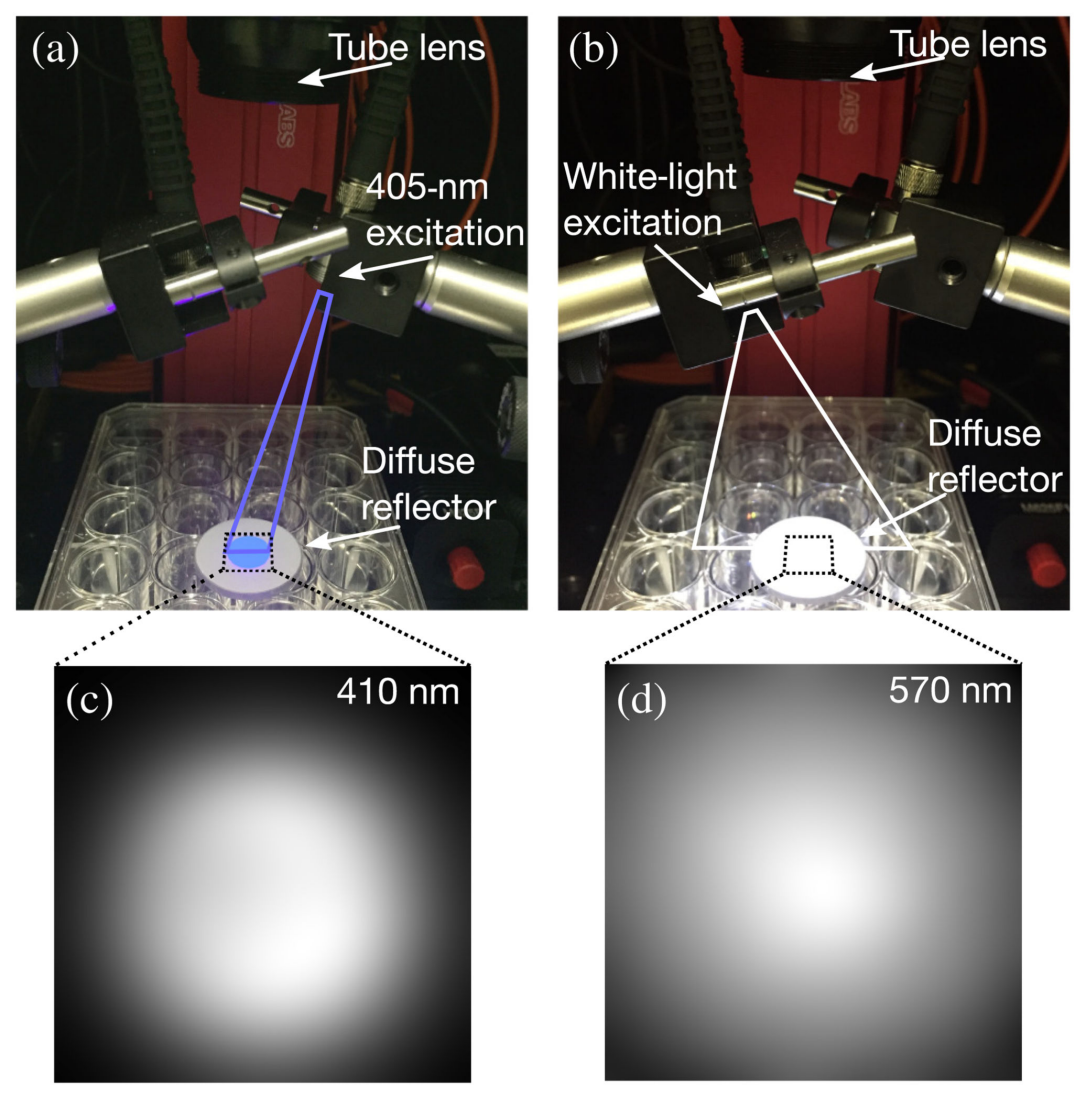
Experimental setup of obtaining reference diffuse reflectances *R*_ref,ex_(*λ*) and *R*_ref,em_(*λ*) under (a) UV excitation and (b) white-light excitation where both the illuminations are not perpendicular to the sample surface. (c) Measured diffuse reflectance at 410 nm on the diffuse reflector showing a heterogeneous distribution over the UV-illuminated area and (d) measured diffuse reflectance at 570 nm illustrating a heterogeneous distribution over the white-light illuminated area.

For precise and reliable quantitative fluorescence imaging, system calibration is an essential step. Typically, the calibration incorporates corrections of undesired background signals (e.g., camera read-out noise, stray light, and instrument offset), instrument spectral responses (camera and LCTF), illumination nonuniformity, etc.^29,30^ However, system calibration is not well-highlighted in the previous reports of using camera-based fluorescence imaging for PpIX assessment in glioma surgery.^21,26^ Therefore, in our model, we particularly calibrate the measurement and compensate for the lighting and collection geometry using normalized diffuse reflectance [$R_{\text{ex}}^{\prime }(\lambda )$ and $R_{\text{em}}^{\prime }(\lambda )$]. In detail, the measured diffuse reflectance *R*_ex_(*λ*) and *R*_em_(*λ*) were first normalized to reference diffuse reflectance *R*_ref,ex_(*λ*) and *R*_ref,em_(*λ*), respectively. The reference diffuse reflectances are measured using a diffuse reflector (DG10-1500, Thorlabs) under the same excitation and measurement settings described previously. Specifically, the *R*_ex_(*λ*) was determined under UV excitation, while the *R*_em_(*λ*) was taken under white-light excitation. The reference diffuse reflectance *R*_ref,ex_(*λ*) and *R*_ref,em_(*λ*) were acquired under UV and white-light excitation for calibration of *R*_ex_(*λ*) and *R*_em_(*λ*), respectively. A measurement setup to illustrate how the *R*_ref_ (*λ*) was acquired, together with two representative reference diffuse reflectance images obtained from the diffuse reflector, is shown in Fig. 2. The two representative reference diffuse reflectance images clearly demonstrate the heterogenous distributions owing to the angular illumination and Gaussian profile light beams. For each pixel in the wide-field image, the measured fluorescence can be expressed as

(3) $$\begin{array}{l} F(\lambda )=(1-{\bar{R}}_{\text{ex}}^{\prime })\left[\frac{f_{\text{PpIX}}(\lambda )}{\mu_{a,\text{ex}}}\right]R_{\text{em}}^{\prime }(\lambda )+\varepsilon ,\;\lambda \in [600,720]\;\text{nm}\\ R_{\text{ex}}^{\prime }(\lambda )=\frac{R_{\text{ex}}(\lambda )}{R_{\text{ref},\text{ex}}(\lambda )},\;\lambda \in [600,720]\;\text{nm}\\ {\bar{R}}_{\text{ex}}^{\prime }≃\frac{1}{3}\sum_{\lambda \in [430;440;450]}R_{\text{ex}}^{\prime }(\lambda )\\ R_{\text{em}}^{\prime }(\lambda )=\frac{R_{\text{em}}(\lambda )}{R_{\text{ref,em}}(\lambda )},\;\lambda \in [600,720]\;\text{nm}, \end{array}$$

where ${\bar{R}}_{\text{ex}}^{\prime }$ is averaged over the normalized diffuse reflectance ${\bar{R}}_{\text{ex}}^{\prime }(\lambda )$ at *λ* ∈ [430,450] nm and *ε* is the measurement noise.

Since the optical absorption of tissue specimen cannot be accurately determined with this system setup and the *μ*_*a*,ex_ of brain tissue and tumor tissue are in the same range from 9 to 17 cm^−1^,^31^ in practice, we neglect *μ*_*a*,ex_ from the model. To retrieve the PpIX concentration, we assume that the fluorescence intensity is linearly proportional to the PpIX concentration; hence, the measured fluorescence can be expressed as

(4) $$\begin{array}{l} F(\lambda )=(1-{\bar{R}}_{\text{ex}}^{\prime }){\left[R_{\text{em}}^{\prime }(\lambda )\right]}^{q}f_{\text{basic}}(\lambda )C+\varepsilon ,\\ \lambda \in [600,720]\;\text{nm}, \end{array}$$

where *C* is the quantitative concentration of PpIX to be retrieved and *f*_basic_(*λ*) is the emission spectrum of PpIX of unit concentration. *q* (*q* = 2.6) is introduced here as a calibration factor similar to the system correction factor that was described in the work of Valdés et al.^21^ This factor is empirically determined in the phantom study when the estimated concentration optimally correlated to the real concentration. Classically, the inverse problem Eq. (4) is solved using least-squares optimization

(5) $$\text{arg}\;\underset{C}{\text{min}}{\left\|\left\{(1-{\bar{R}}_{\text{ex}}^{\prime }){\left[R_{\text{em}}^{\prime }(\lambda )\right]}^{q}f_{\text{basic}}(\lambda )\}\cdot C-F(\lambda \right)\right\|}_{2}^{2}.$$

In this way, we compute a PpIX concentration map *C*(*x, y*)*x, y* ∈ *R*^1024^ over the full field of view of the imaging system.

### 2.5 Total Variation Regularization

While the standard least-squares approach works well in the strong light illumination cases, when the measurement conditions are suboptimal (e.g., low exposure time and low fluorophore concentration), the reconstructed concentration map (*C*) becomes heavily corrupted with noise. To reduce the impact of acquisition noise on the reconstruction noise and thus improve image quality, we propose in this study a reconstruction method appropriate for this application and based on TV regularization. Equation (5) can be rewritten as

(6) $$\text{arg}\;\underset{C}{\text{min}}{\left\|h(\lambda )\cdot C-F(\lambda )\right\|}_{2}^{2}=\text{arg}\;\underset{C}{\text{min}}{\left\|C-\frac{F(\lambda )\cdot h(\lambda )}{||h(\lambda )|{|}^{2}}\right\|}_{2}^{2},$$

with

(7) $$h(\lambda )=(1-{\bar{R}}_{\text{ex}}^{\prime }){\left[R_{\text{em}}^{\prime }(\lambda )\right]}^{q}f_{\text{basic}}(\lambda ).$$

To decrease the influence of the acquisition noise, *L*1-norm TV regularization is now introduced

(8) $$\text{arg}\;\underset{C}{\text{min}}\left\|C-\frac{F(\lambda )\cdot h(\lambda )}{{\left\|h(\lambda )\right\|}^{2}}\right\|1+\delta {\left\|\nabla C\right\|}_{1},$$

where *δ* is the regularization parameter. Equation (8) can be seen as a TV denoising problem on the least-squares solution *C*_ls_

(9) $$C_{\text{ls}}=\frac{F(\lambda )\cdot h(\lambda )}{{\left\|h(\lambda )\right\|}^{2}},$$

and can be solved using a dedicated solver such as the primal–dual algorithm of Chambolle and Pock.^32^ *δ* was chosen for minimizing the differences between the TV-reconstructed concentration map and the ground-truth concentration map; for details refer to Sec. 3.3.

### 2.6 Phantom Experiments

Aqueous phantoms were prepared for evaluation of the imaging system and the reconstruction model, with various concentrations of intralipid (IL) (20% emulsion, Fresenius Kabi, UK) as the scatterer, hemoglobin (Hb) (H2500, Sigma) as the absorber, and PpIX disodium salt (258385, Sigma) as the fluorescent agent.

#### 2.6.1 Evaluation of fluorescence model

**Fig. 3:**
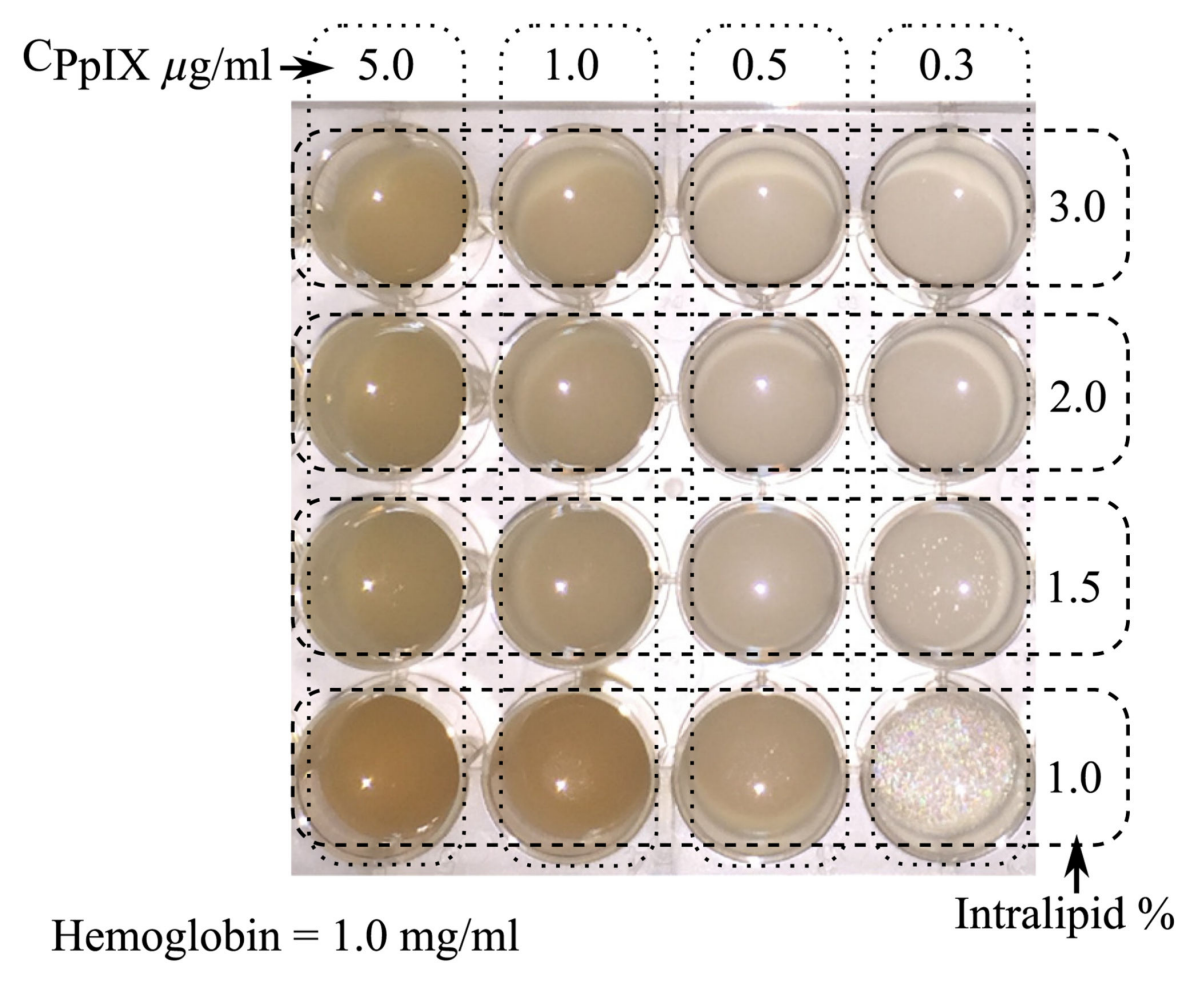
A photograph of phantom set B illustrates that the shade of each phantom in the well varies with the concentration of the PpIX and IL collaterally.

To investigate the influence of the tissue intrinsic optical properties over the PpIX fluorescence emission and assess the impact of absorption and scattering individually, we developed two sets of phantoms (denoted as set A and set B). There were four groups (denoted as A1 to A4 and B1 to B4) in each set, and every group included four phantoms with distinct PpIX concentrations (denoted as e.g., A1,1 to A1,4). Set A had a constant IL concentration of all four groups, while the Hb concentration varied across groups. Conversely, set B shown in Fig. 3 had the Hb concentration kept constant but using different IL concentrations. There were in total 32 phantoms; their composition specifications as well as the corresponding optical properties are shown in Table 1.

#### 2.6.2 Evaluation of system performance

We assessed the performance of the Gen II CMOS camera (ORCA-Flash 4.0) in regards to the sensitivity and accuracy in detecting fluorescence signal under different exposure times in tissue-mimicking optical phantoms. Nine sets of phantoms consisted of combinations of IL concentration of 1.0%, 1.5%, and 3.0% and Hb concentration of 1.0, 1.5, and 2.0 mg/ml. Each scattering-absorption combination was prepared in four different PpIX concentrations of 0.01, 0.05, 0.25, and 1.00 *μ*g/ml, giving in total 36 phantoms. For each phantom, we measured the wide-field images with four different camera exposure times of 10, 20, 50, and 80 ms for each acquisition wavelength, while the other acquisition settings and theconcentration quantification method were kept consistent with the Sec. 2.2.

#### 2.6.3 Evaluation of total variation regularization

To evaluate the impact of TV regularization, a structured phantom incorporating a UCL logo-shaped fluorescent inclusion and nonfluorescent background was developed. The aqueous fluorescent inclusion consisted of 2.0% IL, 2.5 mg/ml Hb, and 0.2 *μ*g/ml PpIX, simulating the optical and fluorescent properties of glioma tissue. For image acquisition, we used 20- and 200-ms exposure times to obtain two series of high- and low- noise acquisitions. A high-noise concentration map (*C*_ls_) and a low-noise concentration map considered as a ground truth (*C*_gt_) were reconstructed from 20- and 200-ms acquisitions, respectively. We then applied TV-based denoising to *C*_ls_ and compared the resulting regularized reconstruction map (*C*_TV_) with the ground truth (*C*_gt_). The contrast-to-noise ratio (CNR) and root-mean-square error (RMSE) were determined to evaluate the improvement provided by TV regularization. The CNR is a quantitative measure of image quality, defined as

(10) $$\text{CNR}=\frac{\mu (\text{ROI})-\mu (\text{Background})}{\sigma (\text{Background})},$$

where the region of interest (ROI) is a 50 × 50 pixel patch area of region of interest in the map, Background is a 50 × 50 pixel patch area at the background region of the map, *μ* denotes the mean, and *σ* denotes the standard deviation. A higher CNR indicates better image quality. RMSE is one of the most commonly used similarity measures that quantify the differences between two images (*X, Y*)^33^

(11) $$\text{RMSE}(X,Y)=\sqrt{\frac{\sum_{i,j=1}^{M,N}{\left[X(i,j)-Y(i,j)\right]}^{2}}{M\times N}},$$

where *M* and *N* are the width and height of the images in pixel, respectively. The smaller the RMSE(*X, Y*), the more similar two images are.

### 2.7 Excised Human Glioma Studies

The use of human surgical tissue in this study was approved by University College London and Epilepsy Society Brain and Tissue Bank (12_SC_0669, ESBTB_MTA_17). Tumor specimens were sampled from patients undergoing 5-ALA-guided glioma resection surgery after obtaining consent, and all the experimental procedures were in accordance with the current UK Human Tissue Authority guidelines. The specimen used in this study was selected and aliquoted from the original excised tumor tissue by a neuropathologist. Immediately after the imaging experiment, the specimens were immersed in 10% paraformaldehyde for fixation and returned to the neuropathology lab for further standard histopathological procedures.

### 2.8 Histopathology

The specimen was sliced at 4 *μ*m. The hematoxylin and eosin (H&E) staining was completed with an automated staining system (Leica ST5020); then the slides were cover-slipped on the Leica CV5030.

### 2.9 Quantitative Correlation

We calculated the correlation coefficient (*r*) and its statistical significance (*p* value for testing the hypothesis of no correlation) to assess the correlation between the quantitative concentration of PpIX (*C*_PpIX_) and the quantitative tumor cellularity of the same region in the specimen. A patch (ROI) of 15 pixel × 300 pixel (short axis × long axis) was extracted from the whole *C*_PpIX_ map; the *C*_PpIX_ was averaged over the short axis providing a concentration profile of the selected patch along the long axis. The cellularity was quantified in this study as nuclear–cytoplasmic ratio (NC ratio) in the H&E histology. For each specimen, the whole H&E-stained slide was scanned at 40× and digitized (Leica SCN400 scanner, Leica Microsystem, UK). Microscopic images of 10× magnification were used to extract the NC ratio. An ROI of 0.2 mm × 2.5 mm (short axis × long axis) corresponding to the position and the size of the *C*_PpIX_ patch was cropped from the whole-slide image. The coregistration between the histology and the *C*_PpIX_ map was achieved by identifying some of the major anatomical landmarks, such as blood vessels and gray–white matter boundaries. The NC ratio was determined in a 0.05-mm window over the region’s long axis of 2.5 mm. The cell nuclei were automatically detected using color-based segmentation available in MATLAB^®^, which transformed the RGB histology images in the *L* * *a* * b* color space and classified violet-blue colored nuclei from the background pink-red color.^34^

## 3 Results

### 3.1 Evaluation of Fluorescence Model

**Fig. 4:**
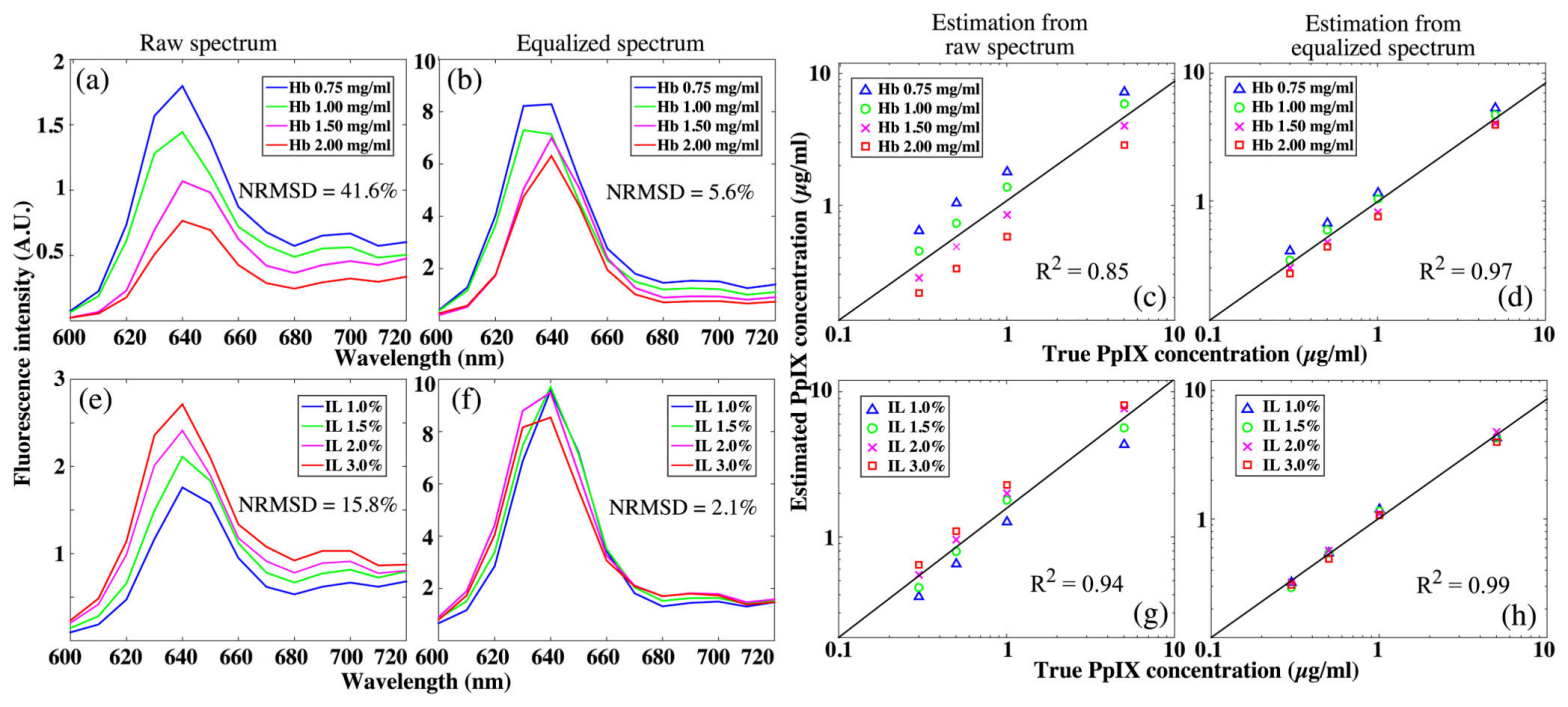
Representative spectrum of phantoms with the same PpIX concentration (0.3 *μ*g/ml) demonstrates diverged emission intensities in (a) Hb-variable group and (e) IL-variable group, with the normalized NRMSD of 41.64% and 15.8%, respectively. The equalized spectra of both phantom groups have unified emission intensity, resulting in markedly reduced NRMSD of (b) 5.6% and (f) 2.1% (f), respectively. For both phantom set A and set B, (d, h) PpIX concentrations that were estimated from Eq. (1) show increased correlation coefficient compared with (c, g) the raw spectra.

The computational model was validated in optical phantoms that featured various scattering and absorption properties as well as different PpIX concentrations. The estimated PpIX concentration map *C*_est_ was reconstructed using Eq. (4) with the measured (data driven) diffuse reflectance, and *C*_PpIX_ was averaged over a 100 × 100 pixels patch in the center of the *C*_PpIX_. As shown in Figs. 4(a) and 4(e), the captured fluorescence emission spectra of phantoms composed of fixed PpIX concentrations but varying Hb (set A) or IL (set B) concentrations featured a notably diversified intensity range, resulting in variations in the estimated PpIX concentration [Figs. 4(c) and 4(g)]. Furthermore, the raw emission intensity at 640 nm was determined to be inversely proportional to the Hb concentration and proportional to the IL concentration [Figs. 4(a) and 4(e)]. The normalized root-mean-square deviations (NRMSD) of the raw peak emission intensity (640 nm) were 41.6% and 15.8% for Hb-variable phantom set A and IL-variable phantom set B, respectively. After applying the model Eq. (1), the emission spectra were equalized as shown in Figs. 4(b) and 4(f), corresponding to reduced NRMSD of 5.6% and 2.1%, respectively. For both phantom sets, the linear fits to the estimated PpIX concentrations that were derived from the equalized spectrum with Eq. (4) presented an improved coefficient of determination (*R*^2^) from 0.85 to 0.97 and from 0.94 to 0.99, respectively [Figs. 4(c), 4(d), 4(g), and 4(h)].

### 3.2 Evaluation of System Performance

**Fig. 5:**
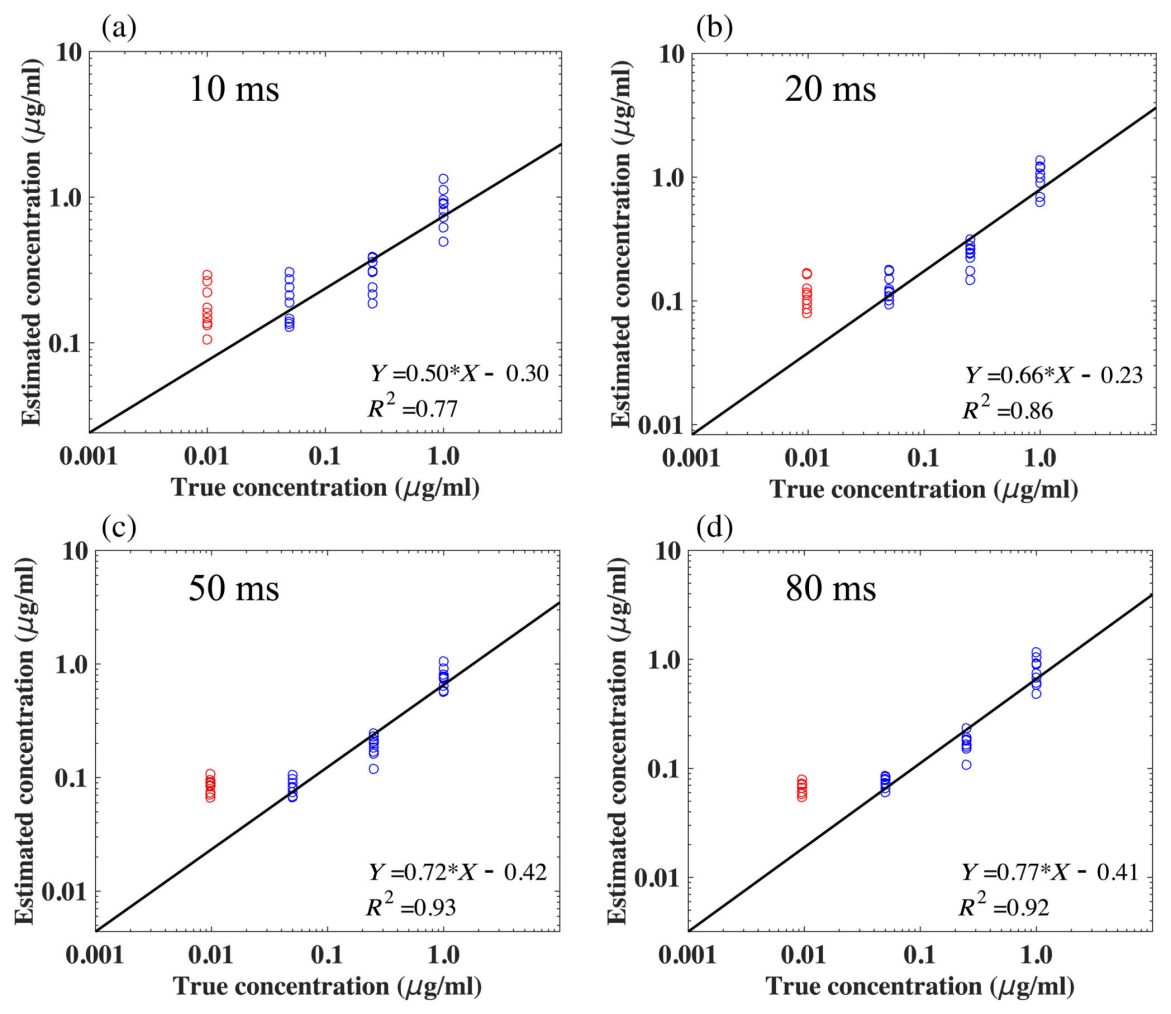
Retrieved quantitative PpIX concentrations against the ground-truth PpIX concentration of nine sets of tissue-mimicking optical phantoms under four different camera exposure times of (a) 10, (b) 20, (c) 50, and (d) 80 ms. Linear fit is performed from PpIX concentration range from 0.05 to 1.00 *μ*g/ml for each plot. Lowest concentration of 0.01 *μ*g/ml is indicated in red as the linearity is broken down in each plot.

We estimated the PpIX concentrations of the nine sets of tissue-mimicking optical phantoms under different camera exposure times, and the quantitative concentration values were averaged over a patch of 50 × 50 pixel in the center of the reconstructed concentration map over the wide-field image. For each exposure time, we plot the retrieved PpIX concentrations against the true PpIX concentrations in Figs. 5(a)–5(d), respectively. At all tested exposure times, the linearity between the estimation concentrations and the true concentration broke down at the lowest concentration of 0.01 *μ*g/ml, which is indicated in red in the respective plots in Fig. 5. This indicates a PpIX detection limit at ~10 ng/ml, which is sufficient for PpIX detection in LGGs.^15^ The coefficient of determination (*R*^2^) of the linear fit was calculated over the concentration range from 0.05 to 1.00 *μ*g/ml. This CMOS system demonstrated an improved sensitivity for PpIX fluorescence detection, with greater *R*^2^ values for exposure times lower than 80 ms ranging from 0.77 to 0.93, compared with the state-of-the-art CMOS-based systems reported in the works of Valdés et al.^26^ and Jermyn et al.,^27^ where the corresponding *R*^2^ values ranged from 0.69 to 0.92. The area of each pixel in the reported EMCCD27 is 256 *μ*m^2^, which is 6 times larger than the used Gen II sCMOS (42.25 *μ*m^2^). Basically, the EMCCD compromises the spatial resolution, using larger pixel size to collect more photons per pixel, thereby yielding a high sensitivity. To provide a fair comparison based on the same total number of pixel, we evaluated the Gen II sCMOS camera’s sensitivity when using the 4 × 4 binning configuration and calculated the respective *R*^2^ values. When using a 512 × 512 pixel frame setting, the detection accuracy of our Gen II CMOS-based system is comparable to the high-sensitive EMCCD system27 for exposure times longer than 20 ms, where the *R*^2^ values both ranged from 0.90 to 0.94 (Table 2).

### 3.3 Evaluation of Total Variation Regularization

**Fig. 6:**
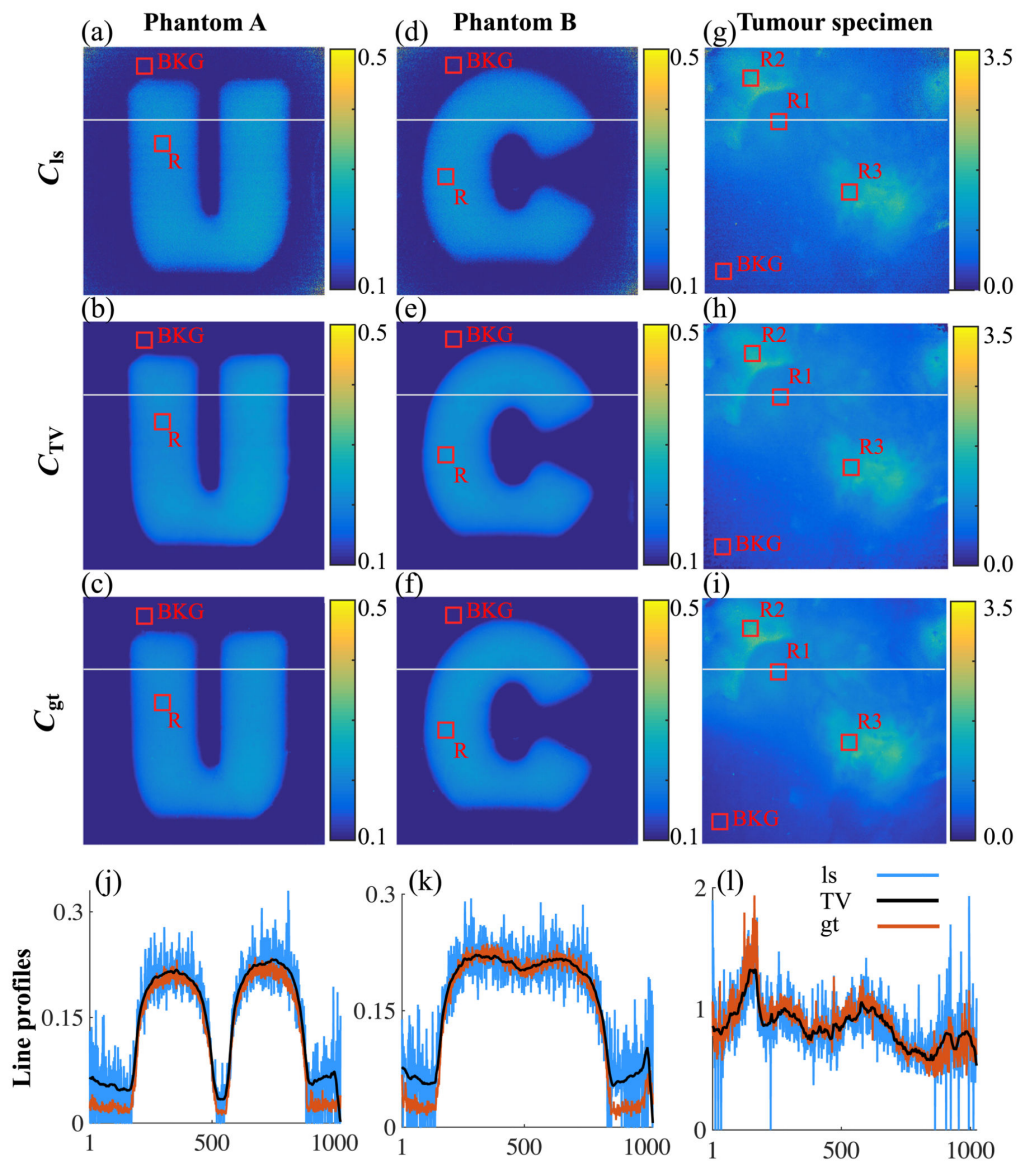
The (b, e, h) results of applying TV regularization/denoising to (a, d, g) noise-contaminated images obtained using low exposure time of 20 ms. Ground-truth images are computed using higher exposure times, (c, f) 200 ms for phantoms and (i) 100 ms for tumor specimen. For each image, noise is reduced/smoothed, edge feature is preserved, and intensity level is retained. Background region (BKG) and regions of interest (R) for each image are denoted in red box. (j, k, l) Intensity profiles of each image demonstrate that TV regularization removes noise, while the edge features and the intensity level/PpIX concentration value are well preserved. Intensity profile is taken from a straight line marked in gray across image’s *x*-axis. ls, least-squares; TV, TV-regularized; and gt, ground truth.

**Fig. 7:**
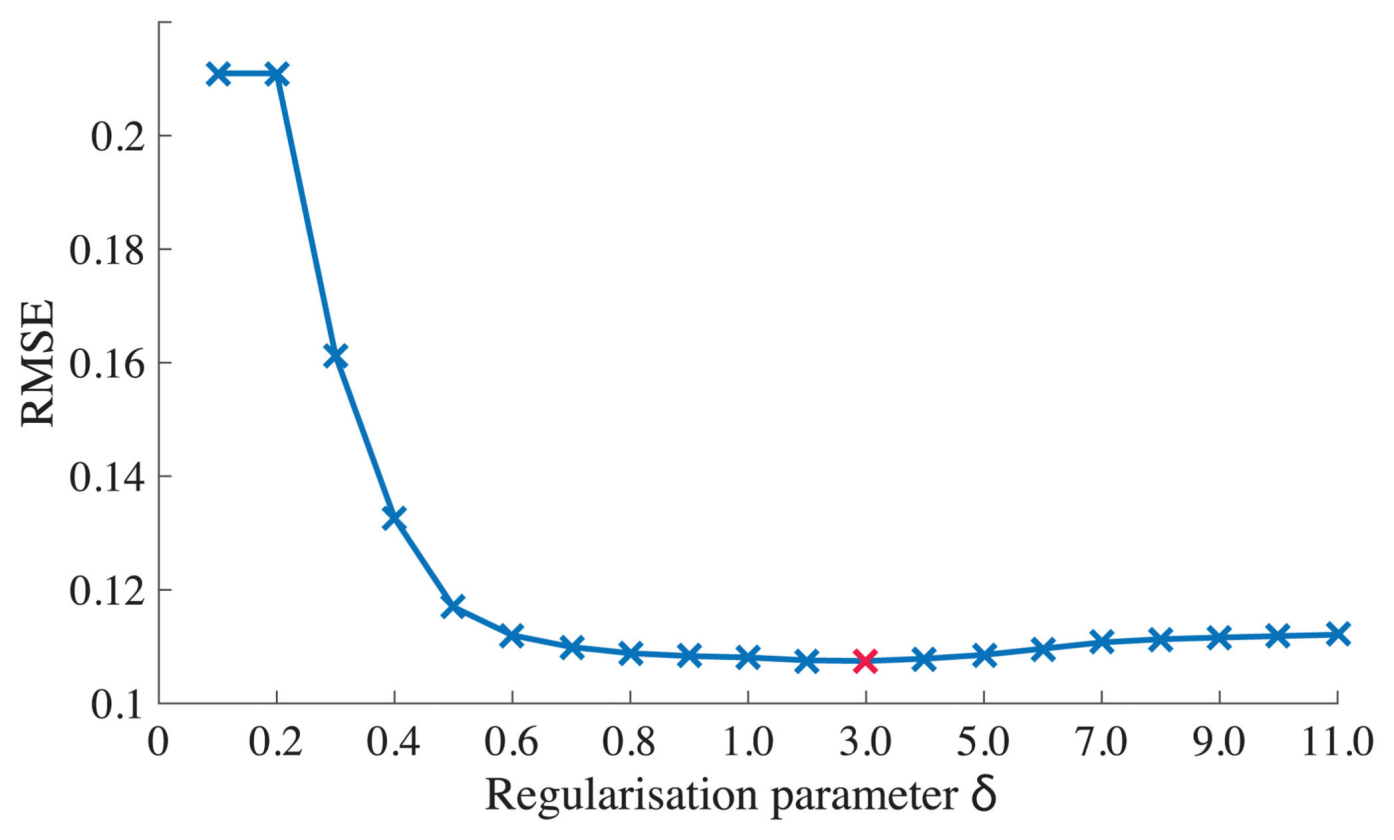
A presentative sample [the tumor specimen shown in the later figure Fig. 6(a)] demonstrating how the regularization parameter *δ* is selected. RMSEs between *C*_TV_ and *C*_gt_ of different regularization parameter *δ* values. The red cross marker indicates that the minimum RMSE locates at *δ* = 3.0.

As stated in Sec. 2.5, *δ* was chosen for minimizing the differences (RMSE) between the TV-reconstructed concentration map (*C*_TV_) and the ground-truth concentration map (*C*_gt_). For example, for the tumor specimen [Fig. 6(g)], we computed the RMSE values using different *δ* ranging from 0.1 to 11.0, plotted in Fig. 7. Based on the RMSE-δ curve, we chose the regularization parameter *δ* = 3.0, as it provided the minimum RMSE value (marked with a red cross in Fig. 7). Notably, the RMSE curve presents a flat shape while *δ* *>* 0.9, suggesting that the regularization results are not highly sensitive to the exact choice of δ. Thus, we selected *δ* = 3.0 for measurements of all other tumor specimens. Likewise, *δ* = 40 was chosen for all phantom experiments.

C_ls_ of phantom A and B and a tumor specimen was constructed from frames acquired in under 20 ms exposure time [Figs. 6(a), 6(d), and 6(g)]. Due to the low camera exposure time, acquired images are contaminated with noise. After processing with TV regularization, image quality was substantially improved by successfully removing noise while the edge features and the intensity level/PpIX concentration value are well preserved [Fig. 6(b), 6(e), and 6(h)]. This is further illustrated in the intensity profiles of each map [Figs. 6(j), 6(k), and 6(l)]. The intensity profile is taken from a straight line marked in gray across image’s x-axis [Figs. 6(a)–6(i)]. The profiles of the *C*_ls_s present prominent random fluctuations. The noisy fluctuations are favorably removed by TV-regularized reconstruction, whereby the concentration values remain comparable to *C*_gt_. The CNR for each concentration map (*C*) is calculated between ROI (R) and background (BKG) marked with a red box, respectively. The CNR values are increased by twofold to threefold after TV regularization and are comparable to the ground truth (Table 3). RMSE between *C*_TV_ and *C*_gt_ is reduced compared with RMSE of *C*_ls_ and *C*_gt_ (Table 3), suggesting that the TV-regularized reconstruction is able to compensate for the loss of the image quality owing to the low exposure time.

### 3.4 *Ex vivo* Human Studies

**Fig. 8:**
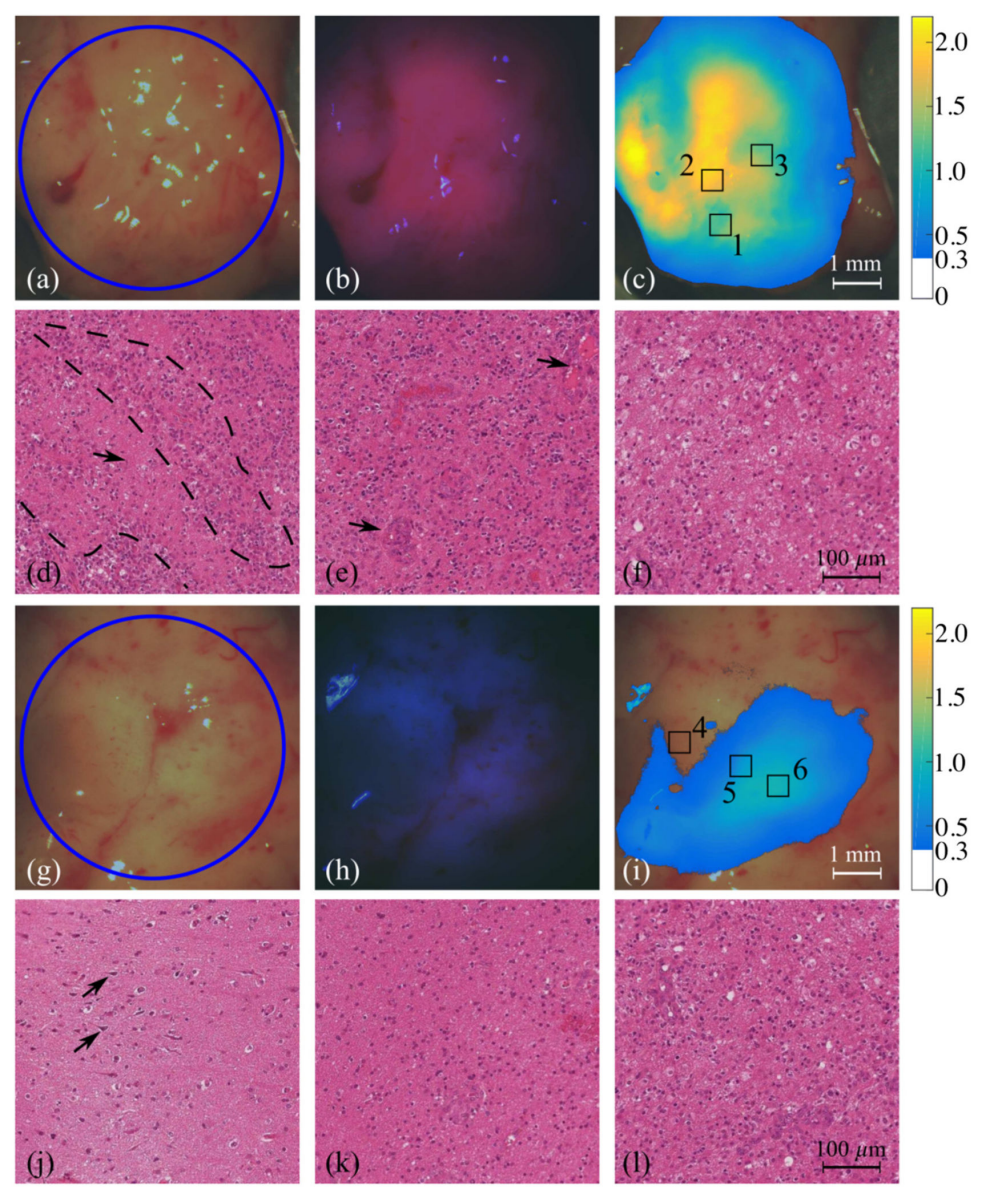
White-light RGB images of two representative tissue areas with blue circles indicating the UV-illuminated area, one of which includes (a) compact tumor tissue, and (g) a combination of infiltration margin and relatively healthy cortical tissue. (b, h) Corresponding fluorescence images demonstrating PpIX fluorescence at *λ* = 640 nm. (c, i) Map of the estimation of concentration of PpIX displaying *C*_PpIX_ *>* 0.3 *μ*g/ml. (d, e, f) Microscopic images of H&E-stained slides, respectively, corresponding to regions of interest 1, 2, and 3 in (c) reveal different typical characteristics of glioblastoma. (j, k, l) Histopathology images of ROI 4, 5, and 6 in (i) display a diffusive proliferation of tumor cells from the margin (k, l) into the relatively healthy tissue (j).

The representative human tumor sample was excised from a glioblastoma multiform (GBM, WHO grade IV) patient and consisted of a large portion of neoplastic tumor tissue, an infiltrated margin, and a small fraction of relatively normal neocortical tissue. Figure 8 shows images of two representative areas, including white-light RGB images, qualitative PpIX fluorescence images, and the quantitative PpIX concentration maps. On the site of neoplastic tissue, strong visible PpIX fluorescence was observed as the vivid red color in the center of the illuminated area and red-violet color in the vicinity [Fig. 8(b)]. Quantitative PpIX con centration map displaying *C*_PpIX_ > 0.3 *μ*g/ml presented a heterogenous distribution across the area [Fig. 8(c)]. The *C*_PpIX_ was around 2.1 *μ*g/ml for vivid red fluorescence, while the red-violet fluorescence corresponded to a *C*_PpIX_ value of 1.2 *μ*g/ml. H&E histology pictures corresponding to three ROIs (500 × 500 *μ*m), where the *C*_PpIX_ featured various different values, are shown in Figs. 8(d)–8(f). In addition to the pathologically increased NC ratio, the variable cellularity in the local area is another typical morphologic feature of GBM. This feature was also revealed in Fig. 8(d) as compact cellularity delineated by a dash line and low cellularity indicated by an arrow; it corresponded to the ROI 1 of which the *C*_PpIX_ showed a “patchy” pattern. Prominent cellularity and endothelial proliferation, both of which were essential microscopic characteristics of glioblastoma [indicated by arrows in Fig. 8(e)], were identified in ROI 2 giving a high *C*_PpIX_ value of 2.05 *μ*g/ml averaged over the region, whereas low cellularity in ROI 3 [Fig. 8(f)] correlated to a moderate *C*_PpIX_ value of 1.36 *μ*g/ml. In contrast, the other representative tissue site consisted of an infiltration margin and a healthy cortical zone [Fig. 8(g)]. Visible but vague fluorescence was detected in the infiltration region, and no visible fluorescence was observed in the healthy region, shown in Fig. 8(h). The estimated *C*_PpIX_ value over the infiltration margin ranged from 0.4 to 0.9 *μ*g/ml, which was markedly lower compared with the tumor dominant area; while in the relatively healthy tissue region, the *C*_PpIX_ was determined to be around 0.15 *μ*g/ml. Normal neurons were able to be identified in the microscopic pictures of ROI 4 [indicated by arrows in Fig. 8(j)], while the neoplastic characteristics became predominant across the tumor margin in ROI 5 and ROI 6 [Figs. 8(k) and 8(l)].

**Fig. 9:**
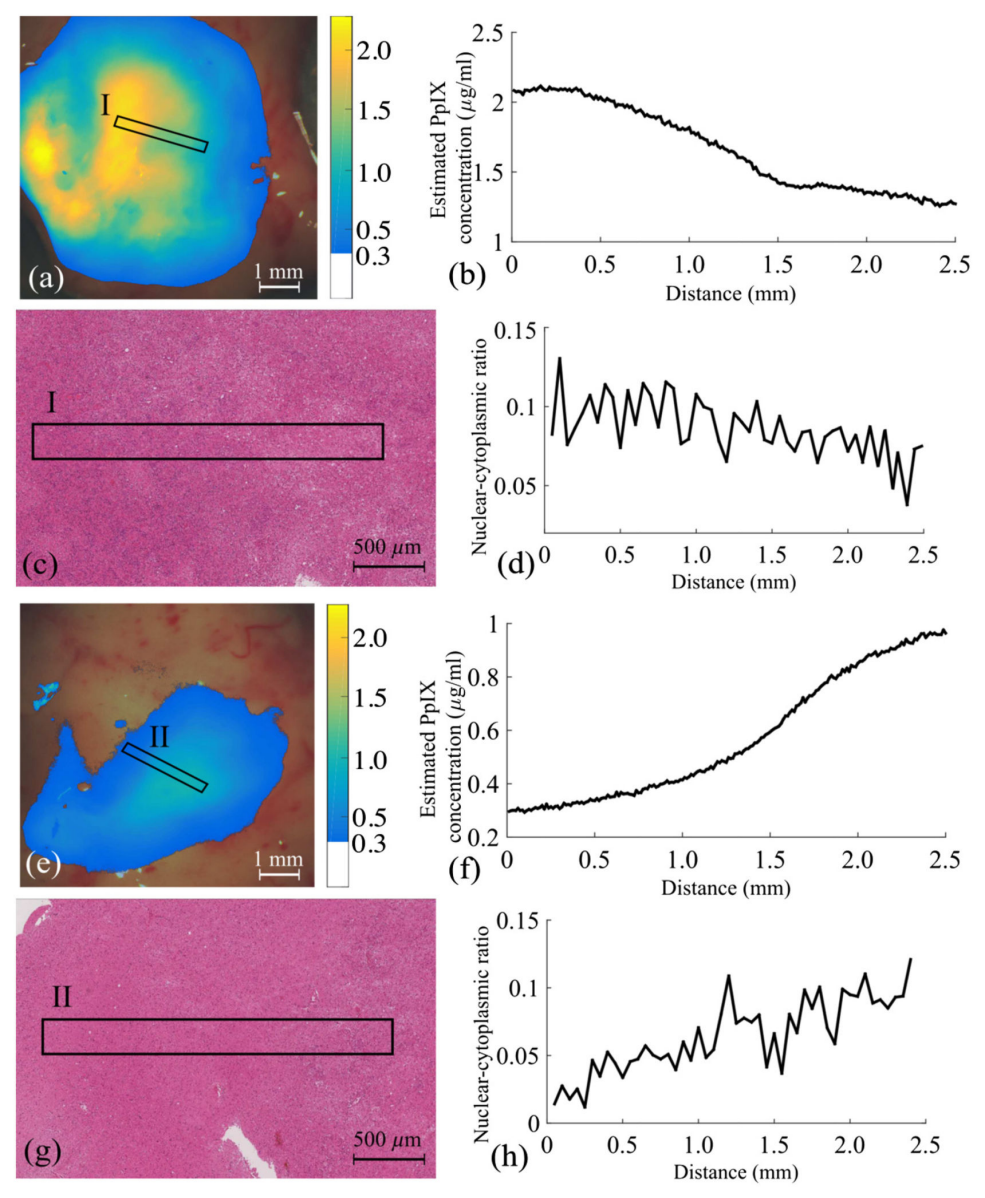
Two ROIs where the quantitative measures in regards to PpIX concentration and cellular density are shown. ROI I and ROI II are indicated in (a, e) the superimposed *C*_PpIX_ maps and in (c, g) the corresponding histology picture, respectively. The *C*_PpIX_ profiles are plotted against the region distance in (b, f), while the NC ratio are presented in (d, h).

We are, to the best of our knowledge, the first to investigate the quantitative comparison between the NC ratio to the quantitative PpIX concentration map retrieved of a wide-field image, in addition to assessing the correlation between PpIX concentration and tumor pathological condition. We computed the *C*_PpIX_ over the short axis of the ROI as a function of the abscissa along the long axis, shown in Figs. 9(b) and 9(f). Similarly, a sliding window (patch size = 0.05 mm) was used to compute the NC ratio as a function of the abscissa along the long axis, shown in Figs. 9(d) and 9(h). The correlation coefficient (*r*) of two representative samples (ROI I and ROI II) demonstrated positive, statistically significant, correlations between *C*_PpIX_ and cellularity NC: ROI I (*r* = 0.63, *p* < 0.05), ROI II (*r* = 0.79, *p* < 0.05).

## 4 Discussions and Conclusions

### 4.1 System Performances

This reported wide-field quantitative fluorescence image system is capable of sensitively detecting PpIX fluorescence in turbid-media and reliably retrieving the truth quantitative PpIX concentration. Its sensitivity is proven to be comparable to the EMCCD-based system.^27^ In addition, the Gen II sCOMS camera features a faster acquisition speed of 200 fps in frame size of 2048 × 1024 at 85-MHz read-out rate, compared with the reported EMCCD^27^ with the highest 63 fps in full frame (512 × 512 pixels) at 20-MHz read-out rate. In this study, the total acquisition and saving time is 26.4 s, which is dominated by data input/output latencies rather than acquisition throughput. We are aware that this is not ideal for implemention in a surgical scenario. However, we are confident that with better computing hardware (for instance using an SSD hard-drive, better CPUs or GPUs, and improved RAM), and with software optimization,^30^ this time can be significantly scaled down (without any compromise on the image quality, resolution, or field of view) to <5 s for acquisition and online processing.

### 4.2 Phantom Studies

The performance of the model on reducing the influence of the tissue intrinsic optical properties on PpIX fluorescence emission was investigated in regards to absorption and scattering, respectively. In general, Hb dominated the absorption at the PpIX excitation band, giving rise to a diminished PpIX emission intensity while the Hb concentration increased [Fig. 4(a)]. Presumably, this was caused by an increased proportion of total incident photons that was absorbed by Hb of higher concentration, lessening the proportion of photons that was absorbed and thus fluoresced by PpIX. Whereas the IL contributes to the scattering across all emission band, IL scatterers also enhance the chances of re-emitted fluorescence photons getting diffusely reflected from the phantom surface and back to the detector [Fig. 4(e)]. Noticeably, the Hb demonstrated a larger influence on distortion of the fluorescence emission compared with IL, characterizing a factor of 3:1 in the NRMSD of peak emission from two representative phantom sets as shown in Figs. 4(a) and 4(e) (NRMSD = 41.6% for the Hb-variable set versus 15.8% for the IL-variable set). As shown in Fig. 11, the data-driven diffuse reflectance at both excitation and emission band corresponded to the model-driven group. However, at 410 nm, there were markedly higher data-driven reflectance intensities [Figs. 11(a) and 11(g)] compared with the simulations [Figs. 11(d) and 11(j)]. This discrepancy was presumably from the specular reflection of the excitation peak (408 nm) when under UV light excitation.

### 4.3 *Ex vivo* Human Tumor Studies

In this work, we focused our tumor tissue imaging feasibility study on GBM: (i) GBM, as it name suggests, has a distinctive “multiform” feature in histopathology, which is expected to be distinguishable in the PpIX concentration map and (ii) the sample consisted of three different tissue regions with respect to pathological conditions (healthy, infiltration, and compact tumor site), which allowed us to make a comparison within one specimen.

**Fig. 10:**
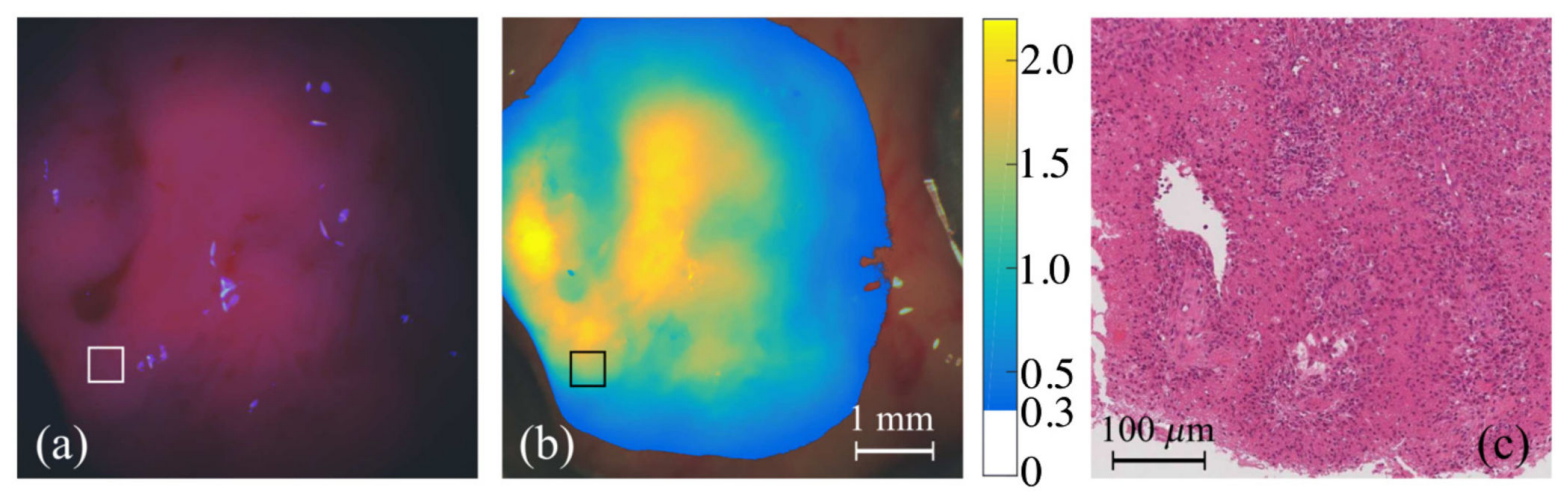
A tumour specimen with region where the fluorescence emission doesn't present strong red colour (a) but corresponded to high PpIX concentration value (b) is later confirmed in histopathology as cancerous site with high cellularity and palisading structures (c). (c) corresponds to area marked with square in (a) and (b).

**Fig. 11:**
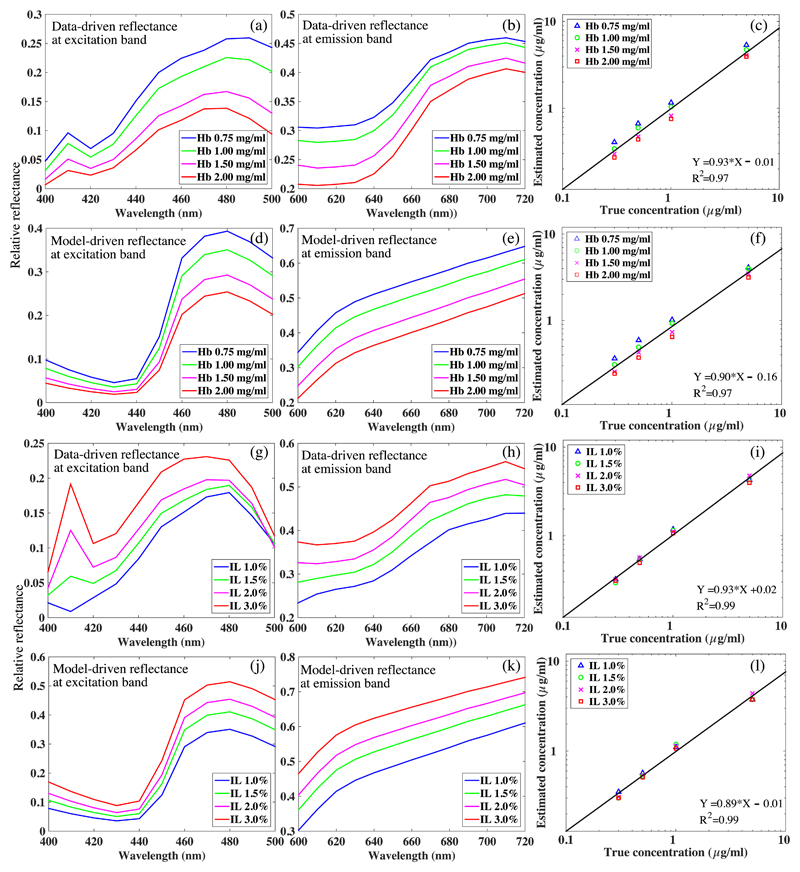
(a, b, d, e, g, h, j, k) The data-driven diffuse reflectance at both the excitation band and the emission band well correspond to the model-driven diffuse reflectance. Accordingly, (c, f, i, l) the PpIX concentrations reconstructed with the measured diffuse reflectance are consistent with the ones determined with the modeled diffuse reflectance, featuring a comparable coefficient of determination of the linear fits.

The specimen consisted of a large portion of white matter, and the tumor sites were primarily embedded in the white matter, whereas a small fraction of tumor tissue was gray matter found on the edge of the specimen. The reconstructed PpIX concentration map essentially indicated the fluorescence emission intensity over the UV-illuminated area. The *C*_PpIX_ map was able to reliably reveal the local histopathological grade in respect to the local NC ratio. Interestingly, some regions where the fluorescence emission did not present strong red color but corresponded to high *C*_PpIX_ were later confirmed in histopathology as tumor site with high cellularity and palisading structures (Fig. 10). As stated in the light propagation theory, the difference between white matter and gray matter with respect to the optical properties probably caused the modulation on the PpIX fluorescence emission. Furthermore, gray matter shows a notably lower optical scattering coefficient at the PpIX emission band compared with the white matter, whereas the absorption coefficients of the two at the PpIX absorption band are in the same range.^35–37^ Based on our findings in phantom studies, with identical PpIX concentration and absorption coefficient, the phantom that had a higher scattering coefficient illustrated stronger peak emission intensity. Presumably, the PpIX fluorescence emission would be diminished in a low-scattering medium and could be recovered to the quantitative PpIX concentration by applying the correction algorithm.

It has been demonstrated that the exogenous 5-ALA causes the synthesis and accumulation of fluorescent PpIX in the mitochondria of epithelia and neoplastic cells.^38^ The PpIX concentration is believed to be proportional to the amount of tumor cells, thus being an indicative value of pathology grades. To date, a few groups have reported their studies on the correlation between PpIX visible fluorescence/concentration and histology/cellularity in brain tumor. Valdés et al.^11^ used PpIX fluorimetry to measure PpIX concentration (*C*_PpIX_) in the excised human glioma specimen and compared its histological score (nontumor, I, II, III, and IV), and they investigated correlations between *C*_PpIX_ obtained *in vivo* in patients using fiber-optic probe and spectrometer to five histopathological categories (control, low grade, high grade, meningioma, and metastasis).^15^ Stummer et al.^23^ correlated visible fluorescence qualities and spectrometric fluorescence to tumor cell density, respectively, where tumor cellularity was assessed using a semiquantitative scale incorporating five classes. Most recently, Lau et al.^24^ demonstrated that qualitative fluorescence intensity and cellularity grade (grades 1 to 4) had strong positive correlations for all tumor groups (glioblastoma, WHO Grade III, and recurrent), with an average Spearman’s correlation coefficient (*r*) value of 0.645. However, to the best of our knowledge, a direct, spatially localized, comparison between *C*_PpIX_ and quantitative tumor cellularity has not yet been reported. We believe that studying whether PpIX concentration spatially correlates to cellularity in brain tumors is of paramount importance for surgical guidance, and our findings would be interesting to readers in this field.

In this study, we elaborated a sensitive quantitative wide-field spectrally resolved fluorescence imaging system and a computational model to retrieve the PpIX concentration from captured hyperspectra images. We evaluated our imaging system and algorithm in a phantom study and demonstrated an accurate estimation of the quantitative PpIX concentration. In an *ex vivo* human tumor study, the PpIX concentration *C*_PpIX_ was estimated for each pixel in the image frame and reconstructed as a quantitative *C*_PpIX_ map over the illuminated tissue area.

We demonstrate that the TV regularization is capable of restoring noise-contaminated *C*_PpIX_ obtained under suboptimal exposure times, thus potentially reducing data acquisition time while keeping a reliable concentration estimation. The *C*_PpIX_ was corroborated to be linearly proportional to the NC ratio; accordingly, it quantitatively correlated to the tumor’s pathological grade. As this is a system development study, a limited number of human samples were included, which could be extended to a larger scale study including different glioma types and grades. To fit in the clinical workflow, instrument miniaturization as well as faster data acquisition would be the future focus.

## Supplementary Material

Appendix

## Figures and Tables

**Table 1:** Phantom compositions and the corresponding optical properties at *λ*_ex_ = 410 nm and *λ*_em_ = 640 nm.

		Concentration			Optical properties			
Phantom		IL (v/v %)	Hb (mg/ml)	PpIX (*μ*g/ml)	$\mu_{\text{s},410}^{\prime }$ (cm^−1^)	$\mu_{\text{s},640}^{\prime }$ (cm^−1^)	*μ*_a,410_ (cm^−1^)	*μ*_a,640_ (cm^−1^)
Set A	A1,1 to A1,4	1.5	0.75	0.3; 0.5; 1.0; 5.0	16.41	5.88	8.14	0.12
	A2,1 to A2,4		1.0	0.3; 0.5; 1.0; 5.0	16.41	5.88	10.85	0.16
	A3,1 to A3,4		1.5	0.3; 0.5; 1.0; 5.0	16.41	5.88	16.28	0.23
	A4,1 to A4,4		2.0	0.3; 0.5; 1.0; 5.0	16.41	5.88	21.71	0.31
Set B	B1,1 to B1,4	1.0	1.0	0.3; 0.5; 1.0; 5.0	10.94	5.88	10.85	0.16
	B2,1 to B2,4	1.5		0.3; 0.5; 1.0; 5.0	16.41	8.82	10.85	0.16
	B3,1 to B3,4	2.0		0.3; 0.5; 1.0; 5.0	21.88	11.75	10.85	0.16
	B4,1 to B4,4	3.0		0.3; 0.5; 1.0; 5.0	32.83	17.63	10.85	0.16

**Table 2:** Comparison of *R*^2^ values across cameras used in quantitative fluorescence imaging systems.

	Camera types				
	
	CMOS^27^	EMCCD^27^	CCD^26^	Gen II sCMOS 4 × 4 binning	Gen II sCMOS 2 × 2 binning
					
Exposure time (ms)	Coefficient of determination of the linear regression (*R*^2^)				
10	0.69	0.91		0.86	0.77
20	0.79	0.93		0.90	0.86
50	0.92 (40 ms)	0.93 (40 ms)	0.91	**0.94**	0.93
80	0.95	0.92		0.92	0.92

This bold number represents the highest number among the number in this raw (exposure time 50 ms).

**Table 3:** CNR and RMSE for evaluation of TV regularization.

		CNR			RMSE	
		*C*_ls_	*C*_TV_	*C*_gt_	*C*_ls_, *C*_gt_	*C*_TV_, *C*_gt_
Phantom A		9.6	34.5	28.9	0.056	0.038
Phantom B		10.5	32.0	28.6	0.052	0.033
Tumor specimen	R1	12.3	23.0	28.0	—	—
	R2	17.1	27.8	32.8	0.21	0.11
	R3	15.9	26.8	31.8	—	—

Note: ls, least-squares; TV, TV- regularized; and gt, ground truth.
