# Supplementary material for: Wide-field spectrally resolved quantitative fluorescence imaging system: toward neurosurgical guidance in glioma resection
Source: J Biomed Opt. Author manuscript; Available in PMC 2019 Sep 12. (PMC6742512; doi:10.1117/1.JBO.22.11.116006)
Supplement: Appendix [file EMS84007-supplement-Appendix.pdf]

Appendix: Model-Based Reflectance in Controlled Phantom Studies

Since the model corrects for tissue scattering and absorption based on the measured-then-normalized diffuse reflectance $R'_{\mathrm{ex}}(\lambda)$ and $R'_{\mathrm{em}}(\lambda)$ (referred to as data-driven diffuse reflectance), it is essential to validate the proposed data-driven diffuse reflectance by comparing with a model-driven diffuse reflectance $R_{\mathrm{mol}}(\lambda)$. We addressed this issue in the phantom study where the absorption coefficient $\mu_a(\lambda)$ and scattering coefficient $\mu'_s(\lambda)$ can be calculated (see Sec. 2.6.1). The wavelength-dependent diffuse reflectance $R_{\mathrm{mol}}(\lambda)$ can in this context be modeled by the following equation,[19] where dependence on λ has been omitted for simplicity reasons:

$$R_{\mathrm{mol}} = \frac{a'}{1 + 2k(1 - a') + (1 + 2\ k/3)\sqrt{3(1 - a')}}, \tag{12}$$

where a' is the reduced albedo and k is the internal reflection parameter that describes the refractive index mismatch between the tissue n_{tissue} and external medium n_{external} (air, in this study)

$$a' = \mu'_{\mathrm{s}}/(\mu_{\mathrm{a}} + \mu'_{\mathrm{s}}) \quad k = (1 + r_{id})/(1 - r_{id}), \tag{13}$$

where the r_{id} can be empirically defined as

$$r_{id} = -1.44 n_{\mathrm{rel}}^{-2} + 0.71 n_{\mathrm{rel}}^{-1} + 0.668 + 0.0636 n_{\mathrm{rel}}$$
$$n_{\mathrm{rel}} = \frac{n_{\mathrm{tissue}}}{n_{\mathrm{external}}}. \tag{14}$$

As introduced above, in the case of known optical coefficients, the model-driven diffuse reflectance can be determined using Eq. (12). For the phantoms used in this study, the reduced scattering coefficient contributed by IL can be approximated by[39,40]

$$\mu_{\mathrm{s}}(\lambda)(\mathrm{cm}^{-1}) = 1.17 \times 10^9 \times \lambda^{-2.33}$$
$$g = 2.25\lambda^{-0.155}$$
$$\mu'_{\mathrm{s}}(\lambda)(\mathrm{cm}^{-1}) = \mu_{\mathrm{s}}(1 - g). \tag{15}$$

The absorption coefficient dominated by Hb at given concentration $C_{\mathrm{Hb}}(\mathrm{mg/ml})$ can be modeled by[41]

$$\mu_a(\lambda)(\mathrm{cm}^{-1}) = [2.303 \times e(\lambda) \times C_{\mathrm{Hb}}]/64{,}500, \tag{16}$$

where $e(\lambda)$ is the molar extinction coefficient.

With known concentrations of IL and Hb, the reduced scattering coefficient (μ'_{s}) and the absorption coefficient (μ_{a}) were determined by Eqs. (15) and (16), respectively. As such, the diffuse reflectance spectra were modeled by Eqs. (12)–(14) and compared with the measured diffuse reflectance spectra for evaluation. The data-driven diffuse reflectance at both the excitation band and the emission band corresponded to the model-driven diffuse reflectance as shown in Figs. 11(a), 11(b), 11(d), 11(e), 11(g), 11(h), 11(j), and 11(k). As a result, the PpIX concentrations reconstructed with the measured diffuse reflectance were consistent with the ones determined with the modeled diffuse reflectance, featuring a comparable coefficient of determination of the linear fits in Fig. 11(c), 11(f), 11(i), and 11(l).
